# Supplementary material for: A UMLS-based spell checker for natural language processing in vaccine safety
Source: BMC Med Inform Decis Mak. 2007 Feb 12;7:3. doi: 10.1186/1472-6947-7-3 (PMC1805499; doi:10.1186/1472-6947-7-3)
Supplement: Additional file 13 — RAP application directory. Sets up directory for RAP (RDF application for PHP) source code [file 1472-6947-7-3-S13.gz › rap/doc/phpdoc/resModel/ResProperty.html]

xml version="1.0" encoding="iso-8859-1"?


Docs For Class ResProperty


## Class ResProperty

Description

Description |
Methods (details)

An RDF Property.

History:

- access: public
- author: Daniel Westphal <at d-westphal dot de mail at d-westphal dot de>
- version: V0.9.1

Located in /resModel/ResProperty.php (line 19)

```
Object
   |
   --Node
      |
      --Resource
         |
         --ResResource
            |
            --ResProperty
```

Method Summary

Description |
Methods (details)

ResProperty
ResProperty
(string $uri)

Methods

Description |
Methods (details)

Constructor ResProperty (line 29)

Constructor You can supply a URI

- access: public

ResProperty

ResProperty
(string $uri)

- string
  $uri

#### Inherited Methods


Inherited From ResResource

> ResResource::ResResource()  
> ResResource::addProperty()  
> ResResource::equals()  
> ResResource::getAssociatedModel()  
> ResResource::getIsAnon()  
> ResResource::getProperty()  
> ResResource::hasProperty()  
> ResResource::listProperties()  
> ResResource::removeAll()  
> ResResource::removeProperties()  
> ResResource::setAssociatedModel()  
> ResResource::setIsAnon()  
> ResResource::setURI()

Inherited From Resource

> Resource::Resource()  
> Resource::equals()  
> Resource::getLabel()  
> Resource::getLocalName()  
> Resource::getNamespace()  
> Resource::getURI()  
> Resource::toString()

Inherited From Object

> Object::toString()

Documentation generated on Thu, 7 Jul 2005 13:43:46 +0200 by phpDocumentor 1.3.0RC3
